# Reflections on Putting AI Ethics into Practice: How Three AI Ethics Approaches Conceptualize Theory and Practice

**DOI:** 10.1007/s11948-023-00443-3

**Published:** 2023-05-26

**Authors:** Hannah Bleher, Matthias Braun

**Affiliations:** Chair of Social Ethics and Ethics of Technology, University of Bonn, Rabinstraße 8, 53111 Bonn, Germany

**Keywords:** Theory–practice gap, Aligned ethics, Ethics by design, Value sensitive design, Embedded ethics, Critical theory

## Abstract

Critics currently argue that applied ethics approaches to artificial intelligence (AI) are too principles-oriented and entail a theory–practice gap. Several applied ethical approaches try to prevent such a gap by conceptually translating ethical theory into practice. In this article, we explore how the currently most prominent approaches of AI ethics translate ethics into practice. Therefore, we examine three approaches to applied AI ethics: the embedded ethics approach, the ethically aligned approach, and the Value Sensitive Design (VSD) approach. We analyze each of these three approaches by asking how they understand and conceptualize theory and practice. We outline the conceptual strengths as well as their shortcomings: an embedded ethics approach is context-oriented but risks being biased by it; ethically aligned approaches are principles-oriented but lack justification theories to deal with trade-offs between competing principles; and the interdisciplinary Value Sensitive Design approach is based on stakeholder values but needs linkage to political, legal, or social governance aspects. Against this background, we develop a meta-framework for applied AI ethics conceptions with three dimensions. Based on critical theory, we suggest these dimensions as starting points to critically reflect on the conceptualization of theory and practice. We claim, first, that the inclusion of the dimension of *affects and emotions* in the ethical decision-making process stimulates reflections on vulnerabilities, experiences of disregard, and marginalization already within the AI development process. Second, we derive from our analysis that considering the dimension of *justifying normative background theories* provides both standards and criteria as well as guidance for prioritizing or evaluating competing principles in cases of conflict. Third, we argue that reflecting the *governance* dimension in ethical decision-making is an important factor to reveal power structures as well as to realize ethical AI and its application because this dimension seeks to combine social, legal, technical, and political concerns. This meta-framework can thus serve as a reflective tool for understanding, mapping, and assessing the theory–practice conceptualizations within AI ethics approaches to address and overcome their blind spots.

## How to Bridge the Theory–Practice Gap in AI Ethics?

In response to recent progress and successes in artificial intelligence (AI), there is an ongoing debate on how to put AI ethics into practice. Whereas in the early days of the debate, the main issue was defining which high-level principles could provide orientation for the ethics of AI, in recent times, criticism of an overly principles-based approach is increasing. Criticism results from the fact that numerous principles-based ethical guidelines concerning the development of AI have been launched in the last years: Principles-oriented checklists, ethics canvases, and evaluative measures seek to advance the implementation of ethics into AI practices and technologies in order to guarantee ethical AI (see, for example, the data ethics canvas of the Open Data Institute [2021], the iRights Lab’s handout (Puntschuh & Fetic, 2020), or the 12-step guide by the World Economic Forum [Madzou & MacDonald, 2020]). Tech giants such as Google, Apple, Amazon, Meta, and Microsoft also developed guidelines and checklists for ethical AI (see, for example, Google AI [2021] and Microsoft’s Fairness Checklist [Madaio et al., 2020]). Criticism of such principles-based approaches warns that principles alone cannot guarantee ethical AI. For example, AI ethics is criticized for being ethics-washing (Hao, 2019) but not operationalized and hence, it is suggested that AI ethics can even be regarded as useless (Munn, 2022). Instead, there is a need for standardized practices, and proven methodologies for ethical AI engineering, besides common aims, a professional history, robust legal structures, and professional accountability mechanisms (Mittelstadt, 2019). The intertwining of ethical theory and practice thus becomes central to the scientific and public debates on AI ethics. Or—as Morley et al. (2020) put it—focusing on the translation between the *what* and the *how* of AI ethics is key.

On a conceptual level, these criticisms point to a theory–practice gap in AI ethics. Such a gap can be generally understood as a mismatch between the theoretical discussion and the real practices of engineering AI. As Schiff et al. (2021) point out, there can be many reasons for a theory–practice gap, such as a lack of incentives to engage ethically, an over-abundance of tools, the complexity of AI systems, the problem of many hands followed by the question of who should take the ethical lead, or the disciplinary divide between ethics and engineering, or the general lack of resources and established methods, structures, and tools in an organization to manage ethical AI engineering. There are different current accounts on how to define such an appointed gap. Mostly, this gap is understood as a lack of putting theory into practice. There is a variety of ways to locate the specific lack, for example, it is described as a lack of operationalization (Hao, 2019), a lack of regulation (de Laat, 2021), or a lack of translation (Morley et al., 2020). This paper understands the theory–practice gap in a conceptual sense, meaning that we look at the entanglement of these three types of lacks.

This article examines, therefore, how the theory–practice gap is addressed within current approaches to AI ethics. Through this exploration, this article aims to provide a meta-framework as a reflective tool for understanding, mapping, and assessing the conceptualization of theory and practice within AI ethics approaches. In a first step, we will focus on three prominent approaches: the *embedded ethics approach*, an *ethically aligned approach*, and the *Value Sensitive Design (VSD) approach*. Of course, these three approaches do not represent the entire spectrum of applied approaches to AI ethics, but they illustrate conceptual foci on how to bridge the gap identified. Further—as we will analyze in this article—there are different understandings of what is meant by theory and practice. In a second step, therefore, we demonstrate that all three approaches perceive theory and practice differently. Accordingly, the approaches have different strengths and weaknesses: an embedded ethics approach is strongly oriented toward its application context but risks being biased by it; ethically aligned approaches are principles-oriented but lack justification theories to deal with trade-offs between competing principles; and the interdisciplinary VSD approach is based on stakeholder values but needs to be linked to political, legal, or social governance aspects. Against the backdrop of this analysis and critical theory, in a third concluding step, we develop a meta-framework as reflective tool. For this, we propose three reflective dimensions to better conceptualize theory and practice in applied AI ethics approaches as well as to address and overcome their blind spots. We claim, first, that the inclusion of the dimension of *affects and emotions* in the ethical decision-making process stimulates reflections on vulnerabilities and experiences of disregard as well as marginalization. Second, we state that the dimension of *justifying normative background theories* provides both standards and criteria as well as guidance for prioritizing or evaluating competing principles in cases of conflict. Third, we argue that reflecting the governance dimension in ethical decision-making is an important factor to reveal power structures as well as to realize ethical AI and its application because this dimension seeks to combine social, legal, technical, and political concerns.

## How Theory and Practice are Conceptualized Within AI Ethics

In AI ethics, different approaches currently try to overcome the theory–practice gap. In this section, we examine in more detail how these approaches conceptualize theory and practice, as well as their interrelation. For this purpose, an embedded ethics, an ethically aligned, and a Value Sensitive Design (VSD) approach to AI ethics are contrasted. These approaches obviously do not represent the full range of applied AI ethics approaches. But each approach exemplifies different conceptual priorities for certain elements, chances and shortcomings, or perspectives of ethical reflection: While a bottom-up embedded ethics approach tries to incorporate ethics qua persona into the technological context (McLennan et al., 2020, 2022), an ethically aligned approach, on the contrary, builds on politically discussed and well established principles (Morley et al., 2020); whereas a VSD approach identifies values in the technological design process and seeks to integrate and implement values into the context of technological development (Friedman et al., 2013).

For our analysis, we use basic methods from coherence theory in order to understand how these three approaches conceptually link theory and practice (Sugarman & Sulmasy, 2001). One central method in this regard is the wide reflective equilibrium approach (Daniels, 2020). This abductive method combines deductive and inductive analysis elements to gain a more in-depth understanding of a situation, problem, or an issue. Here, abductive means alternating back and forth between both inductive and deductive elements. For our analysis, this abductive method means that first, we hermeneutically explore the main conceptual foci by outlining the understanding of theory and practice. In addition, we discuss the interrelation of theory and practice within each approach to gain insights into their conceptual strengths and weaknesses. By this, we lay the foundation for a meta-framework that aims to address and overcome the blind spots of current AI ethics approaches and their theory–practice conceptualizations. Along these lines, we begin the analysis with a brief overview of each approach, then we examine each approach according to three guiding questions: (1) How is practice understood? (2) How is theory understood? (3) How is the relation between theory and practice understood and conceptualized? Table 1 maps the results of this analysis.

**Table 1:** Results of the conceptual analysis of three AI ethics approaches: embedded ethics, ethically aligned approach, value sensitive design

Approach	Embedded ethics	Ethically aligned approach	Values sensitive design
How is *practice *understood?	Specific AI engineering or research process defined by situation-specific ethical issues	Application of ethical tools in design process	Multidimensional design process
How is *theory *understood?	Set of professional ethical judgments	Set of ethical principles	Set of design inherent values
How is the *relation between theory and practice* understood and conceptualized?	Involved ethics experts and stakeholders ethically reflect on specific ethical questions and issues	Tools transfer principles to practice	Design requirements transfer values into the design

## Embedded Ethics

As a first step, we outline the so-called embedded ethics approach. Not many AI ethics approaches refer to themselves as embedded ethics approaches, therefore, this article takes a hermeneutical narrative account to sketch this approach. Embedded ethics in the field of AI is mostly addressed in an education-oriented engineering context (Grosz et al., 2019; Zuber et al., 2022). In this context, it is referred to as an approach for teaching ethics in engineering curricula (Bogina et al., 2022; Li & Fu, 2012). This means, for example, that ethics modules are integrated into engineering courses, or interdisciplinary reflection or deliberation is embedded into engineering processes. These approaches aim to embed ethics into AI engineering processes by providing ethical training for engineers. Thus, this kind of virtue-ethical approach focuses narrowly on the engineer’s role in the process. Not only engineers and their ethical awareness, however, can be in focus of an embedded ethics approach but also ethics experts themselves who are integrated into the AI engineering process: for example, when big tech companies hire ethics experts and integrate them into their AI engineering teams. These phenomena and approaches, while more and more common, have not yet been labeled as embedded ethics.

Following this brief overview of embedded ethics, the question arises of who is perceived to be responsible to embed ethics into practice. In addition, a second issue inquires what it means to embed ethics into practice. In other words, the conceptualization of embedding ethics is in question. In general, an embedded ethics approach characterizes itself by being bottom-up and inductive, oriented toward the engineering process of technologies and the moral intuitions and knowledge of the stakeholders involved. However, this can only be a broad description. Since the label of embedded ethics has only recently emerged in the discussion, there are not that many approaches that label themselves as an embedded ethics approach. However, to address such conceptual questions, this paper focuses in the following on the most prominent approaches of McLennan et al. (2020, 2022) and Fiske et al. (2020) as the latest embedded ethics conceptions in the field of AI. These approaches serve as illustrative examples of embedded ethics, whose conception of theory and practice also applies in its basic characteristics to the above-mentioned education-oriented approaches.

As an interdisciplinary team, McLennan et al. (2020, 2022) propose an applied ethics account that attempts, as they state, to integrate social, ethical, and legal considerations in a deeply integrated as well as collaborative manner into the overall technological development processes of AI. Within this approach, ethicists as dedicated members of the AI development team bring the ethical perspective—in the authors’ words—“to the workbench” (McLennan et al., 2020). The task of these ethics experts, as described by the authors, consists of regular, iterative, and continuous processes of ethical reflection, collaborative ethical exchange in the development and design team, ad hoc suggestions on acute ethical issues, and the clarification as well as explanation of ethical dilemmas and issues to the tech workers (McLennan et al., 2020).

Another article further specifies the embedded ethics approach: Fiske et al. (2020) conceptually and methodically represent the embedded ethics approach as a complement to the ‘pipeline model’ (Char et al., 2020). The pipeline model proposes a framework to identify ethical issues along the developmental pipeline from conception to implementation, for example, of a Machine Learning Health Care Application (ML-HCA), combined with a parallel pipeline of evaluation and oversight (Char et al., 2020). Along these pipelines, ethical key questions are raised to uncover ethical issues. Char et al. (2020) highlight the limitations of this model, pointing to caveats such as its conceptual imperfection, the unresolved question of who is responsible for ethical development, and the remaining problem of how to resolve trade-offs between ethical considerations. This is exactly where the embedded ethics approach of Fiske et al. (2020) comes into play by offering solutions to these caveats. In doing so, Fiske et al. (2020) sharpen their approach by pointing out that, firstly, the embedded ethics approach aims precisely at incompleteness of ethical considerations. In other words, the embedded ethics approach embraces incompleteness by being practice-oriented rather than prescriptive, as the authors state (Fiske et al., 2020). Second, regarding the question of responsibility, they consider ethicists as responsible for the ethical reflection of AI development, and third, they encourage a process of evaluating trade-offs.

As this overview of the embedded ethics conception by McLennan et al. (2020, 2022) and Fiske et al. (2020) already indicates, the authors understand practice in relation to the technological AI development process. The practical “workbench”, as referred to, is the AI engineering process. Here, ethical reflection is embedded as a regular, context-sensitive process. This points to a fundamental assumption of such embedded ethics approaches, namely that it is assumed that innovative and exploratory practice always raises ethical issues, and new ethical concerns arise in the specific engineering process. From an ethical perspective, this may sound self-explanatory at first, but for the understanding of practice, it can be deduced that specific situational ethical issues characterize engineering practices. A reported case study from McLennan et al. (2020) reinforces this understanding that the technological engineering process with its specific emergent issues is the benchmark for interdisciplinary ethical consultations. Succinctly, situational ethical problems of the technological development process determine what qualifies as a concrete practice to which such an embedded ethics approach refers.

In terms of the understanding of theory, in an embedded ethics approach, as the name indicates, the ethical theory is intended to be embedded into practice. Within the conception of McLennan et al., (2020, 2022) and Fiske et al. (2020), this embedding of theory is ensured by an ethicist as a member of an interdisciplinary team. This indicates that the inclusion of ethicists in the engineering process is almost synonymous with the embedding of ethical theory in practice. However, according to the authors, an ethicist is a person who is appropriately trained, has ethical knowledge, and can demonstrate professional competence. Professional education, ethical knowledge, and training of the ethicist represent subsequently ethical theory-building. Thus, in this context, theory is, first and foremost, perceived as a profession that can be integrated qua persona into a specific ethical situation. Through their professional ethical judgments the involved ethicists thus bring ethical theory to the “workbench”.

Within this embedded ethics approach, theory and practice merge through the inclusion of ethicists given a specific ethical issue. The case study reported by McLennan et al. (2020) further implies that the theoretical-ethical reflection on practical questions or dilemmas takes place as interdisciplinary workshops with all team members of a technological development or research process. It is precisely these joint events in which all involved stakeholders take up ethical reflections and consider ethical issues in a multi-perspective, professionally guided manner. It is, thus, the involved stakeholders who conceptually bridge theory and practice in these interdisciplinary workshops by reflecting on ethical issues. Again, qua persona, in the persons of the involved stakeholders, theory and practice are conceptually delineated.

To summarize and critically appreciate this account, first, a very specific practical ethical issue is assumed that can arise at any time during the design process. McLennan et al. (2020, 2022) emphasize with this understanding a non-prescriptive ethical approach to practice with a distinct bottom-up character of ethical decision-making. This means in the case of the described concepts that the approach is driven by the engineering practice and the emerging issues identified by ethics experts and/or the stakeholders involved and guided by their ethical questions or concerns. Against this background, the relevance of ongoing ethical reflection in the design process is placed at the center of the approach and thus legitimizes the role of ethicists as dedicated team members in AI engineering. As a second central element, ethical theory is understood as professional judgments which are qua persona embedded in the practical context. Although the authors of the approach describe the professional competence of ethicists as accompanied by an ethical qualification, it remains very vague what may be understood by a personal competence of ethical inquiry. In this sense, the ethical approach crucially depends on how that person (or team) develops and conducts ethical theory and practice. In addition to the emphasis on an intuitive bottom-up process for the selection of ethical issues in the design process, ethical decision-making furthermore depends on the subjectivity of ethicists, which may provoke biases. Moreover, this subjectivity-driven setting is also enormously vulnerable to abuse of power, as both ethics experts can exploit their position and not only guide ethical reflection but also manipulate it. But the ethics experts themselves also run the risk of becoming victims of power structures by not being heard with their ethical judgments or having no influence on the AI engineering process. For example, in the setting of tech companies, ethical reflection is in danger to fall victim to power structures driven by the logic of profit orientation. Third, the stakeholders involved prove to be the interface between theory and practice, reflecting on their practical experiences and ethical reflections in an interdisciplinary workshop. The case study described from McLennan et al. (2020) outlines practical experiences with such a bottom-up concept. However, neither the methodological aspects of the ethical reflection process nor the criteria relevant to ethical decision-making are further specified in this conception.

Against this background, we argue that two fundamental questions arise: Firstly, the bottom-up orientation raises the question of which ethical criteria and principles guide ethical decision-making. At least, the authors of the approaches consider well-known ethical issues of AI ethics relevant for ethical reflection (e.g., privacy, transparency, responsibility, etc.). They are mentioned as the basis of the approach and reference is made to overarching principles, which, however, are not explained further, neither conceptually nor methodologically. Interestingly, it is precisely the lack of criteria and benchmarks for ethical judgments that the authors of this conception highlight as an open question: “[…] [H]ow to judge whether embedded ethicists have done a good job[?]” (McLennan et al., 2020). At least, the development of methodological quality measurements for AI ethics is proposed as a solution (McLennan et al., 2020). Secondly, the question arises of how the understanding of theory as a set of professional ethical judgments and qua persona can be conceptually integrated in such a way that biases, subjectivity, and power abuse are prevented or contained. At this point, the lack of guiding principles and justification theories must be noted. As a result, ethical judgments remain without justification and the embedded ethics’ theory–practice concept is thus on shaky ground: Hence, ethical judgments in structurally similar cases can turn out completely differently, with different justifications. This is a pitfall for an inconsistent approach and incoherent ethical judgments. It may cause difficulties in social trust in the approach which could probably be avoided by integrating principles and/or policy considerations.

## Ethically Aligned

In contrast to the bottom-up, stakeholder, and context-oriented embedded ethics approach, ethically aligned approaches to AI are principles-driven. This means ethically aligned approaches emphasize specific, high-level principles such as transparency, responsibility, fairness, human rights, or human well-being as relevant elements to their applied ethical account (IEEE Global Initiative on Ethics of Autonomous and Intelligent Systems, 2019; Vakkuri et al., 2019). Various organizations and bodies have elaborated such principles, to which ethically aligned approaches refer and are oriented (Beijing Academy of Artifical Intelligence, 2019; High-Level Expert Group on Artificial Intelligence, 2019; OECD, 2019).

The ethically aligned approach can differ widely in its applied conceptualizations. Very prominent are the guidelines of the IEEE Global Initiative on Ethics of Autonomous and Intelligent Systems (2019) for Ethically Aligned Design (EAD). In conceptions like this, in contrast to the embedded ethics approach, specific overarching principles are essential and conceptually combined with tools, technical constructs or instruments, guidelines, or discursive methods of ethical reflection. In the practice of AI engineering, this could then proceed in such a way that tools and methods are selected according to certain principles to initiate an ethically aligned process or to design ethically aligned AI artifacts. The outcome of this approach are ethically aligned processes as well as designs, for instance for autonomous systems (Vakkuri et al., 2019) or assistive robots (Weng & Hirata, 2018), as well as ethically aligned tools, for instance, software models (Jantunen et al., 2021), or unbiased deep learning models (Danner et al., 2021), or tools that control or evaluate ethically aligned processes (Halme et al., 2021). Vakkuri et al. (2021) have developed a more detailed method with their ECCOLA approach, a methodological proposal for implementing ethically aligned AI. This method aims to provide developers with an actionable roadmap for implementing AI ethics. With all their different concerns and objectives, what unites these different efforts is the attempt to put principles straight into practice.

Following the conceptual question of this article, however, we again choose a narrative perspective toward the ethically aligned approach. Therefore, to exemplify and illustrate in more detail what characterizes an ethically aligned approach in a conceptual perspective, we outline the concept of Morley et al. (2020) thoroughly. This concept illustrates the principles orientation of an ethically aligned approach particularly well and paradigmatically. The approach by Morley et al. (2020) is based on a typology that aligns ethical tools with principles of the European High-Level Expert Group on Artificial Intelligence (AI HLEG) (2019), oriented toward each stage of the development process of machine learning (ML). The stage orientation and typology characterize this ethically aligned conception by Morley et al. (2020) very specifically. Other ethically aligned approaches conceptualize their principles orientation in different ways, when they develop, for example, tools to guarantee transparency or software models that prevent discrimination. At first glance, the method and direction of Morley et al.’s conception appear to be top-down, driven by predefined principles—but the authors of this exemplary approach seek to include bottom-up elements such as ongoing discursive and participative processes as well. The idea of this approach is that the typology is used in the ML development process “to enable a shift from a prescriptive ‘ethics-by-design’ approach to a dialogic, pro-ethical design approach.” (Morley et al., 2020) In this sense, the authors proclaim to drive forward a pragmatic version of Habermas’s discourse ethics. Although the approach is principles-oriented, the link to discourse ethics describes the author’s intention not to set morals and norms in a ‘top-down fashion’. In fact, the authors claim that principles emerge from a discursive process (Morley et al., 2020). In highlighting the discursive process, this principles-oriented ethically aligned conception integrates both up- and down-streaming elements in the ethical reflection process.

Practice, in this approach, is essentially described as the *how* of theory. The conception by Morley et al. (2020) illustrates that practice is about applying or testing theory in the real world. The focus on using typologized tools emphasizes, along this line of understanding, that practice specifically relates to the design process of ML. This is also highlighted by the fact that in their typology, the authors align ethical tools with the design process of ML. The application of tools in the design process constitutes, in this sense, the ethical practice of this conception in developing ethically aligned products or algorithms.

Theory, within this conception, is to be understood from its principles orientation. The politically discursively developed principles of the European AI HLEG are, as described by the authors, the starting point of the typology and the ethical reflection within this AI ethics conception. Morley et al. (2020) declare a set of five guiding principles as the leading ethical principles of AI: beneficence, non-maleficence, justice, respect for autonomy, and explicability. Above all, the central role of the principles regarding the understanding of theory becomes clear from the fact that the authors of this approach aim for a pro-ethical concept of ethics. That means that ethics aims at enabling societal agents to choose their actions and behaviors freely but in a way that safeguards the values, the set of principles, and the kind of ethics that society considers fundamental (Morley et al., 2020). Whilst in this approach, dialogical ethical deliberation appears to be central for theory building, the set of principles, however, determines the direction of deliberation. In this sense, theory building is described as situated between a prescriptive approach and a dialogical understanding. In this approach, ethical considerations and judgments are guided by a set of principles that form a deliberatively constituted, theoretical superstructure.

The interrelation between theory and practice, within this conception, is conceptualized by the application of ethical tools in different contexts. The ethical tools themselves can be understood as the connecting means between theory and practice. Applying the tools is described as the conceptual point at which principled, discursive ethical theory merges with practice, namely in the design process. The application of these ethical tools is then the conceptual interweaving of theory and practice, where principles are transferred into practice. To put it another way, the ethically aligned algorithm is the fusion of a set of principles and the design process.

Considering this understanding of theory and practice in comparison to the embedded ethics approach, the principles orientation emerges as central in this conception. Although practice is understood as a design process, similar to the embedded ethics approach, the practical perspective is not setting the tone within the ethically aligned approach. Principles, instead, are the starting point of ethical reflection and ethical design. The understanding of theory, on the other hand, is based on discursive theory building driven by principles. The principles according to Morley et al. (2020) shall serve to avoid harm, to protect human rights, and to enable ethical decision-making.

However, this principles-driven conception also draws attention to a pitfall: There is a lack of justification theories as to why certain principles are applied and others are not. While Morley et al. (2020) state to take a deliberative approach, it is not clear whether or to what extent justification theories or general objectives underpin the selection of principles. At this point, of course, one could object that ethically aligned approaches refer to established principles that are based on political processes in which justifications for the individual principles naturally play a role. Nevertheless, in ethically aligned conceptions the principles are adapted or applied often without their justifications. Explanatory theories, however, in which the ethical principles are embedded, are essential to formulate objectives, to offer guidelines for action, or to provide orientation. Deductive principles-oriented approaches require, in particular, plausible reasoning for ethical judgments by disclosing their criteria in order to justify the appropriateness and coherence of judgments. Especially in conflict situations with competing principles, such as, for example, explicability and justice, it is necessary to justify why claims for justice might outweigh demands for explicability. Hypothetically, this may occur in cases of conflict, for instance, if the disclosure of algorithms was required by law. The economic disadvantage for the provider or designers of an ML-driven software could provoke unfair scenarios and cause damage that is disproportionate to the transparency requirement, for instance, for shopping recommendations or a corresponding data analysis.

Ethical decision-making, therefore, requires a well-founded and plausible framework of justifying reasons. Otherwise, without any justification, principles and their application transferred to tools are either inappropriate, meaningless, or merely an end in themselves. Furthermore, just because a principle proves to be empirically relevant, such as, for example, transparency (Jobin et al., 2019), that does not mean that transparency is equally meaningful in all areas, nor that transparency is always interpreted in the same way. Therefore, solid and pluralism-sensitive arguments and justification frameworks are necessary if principles are to be put into practice.

## Value Sensitive Design

Probably the most prominent approach in engineering ethics of the last 30 years is the Value Sensitive Design (VSD) approach. VSD is an umbrella term for different value-driven approaches (Simon, 2017). It is discussed and applied in various technological fields of innovation for the development of ethical and responsible design, such as within the design of information systems (Brey, 2010; Friedman et al., 2013) and human-computer interaction (Borning & Muller, 2012), as well as in energy projects (Dignum et al., 2016), sensor technology (Dechesne et al., 2013), augmented reality (Friedman & Kahn, 2000), nanotechnology (Timmermans et al., 2011) or robotics (van Wynsberghe, 2013, 2016) and many others—just as nowadays for AI technologies (Simon et al., 2020; Umbrello, 2019).

In engineering ethics, VSD is a specific approach to defining ethical values throughout the entire technological design process (Friedman et al., 2013). The approach aims at an ethical ‘translation process’: core values are translated into concrete standards which are further translated into concrete design requirements. Following a tripartite methodology, the design process is considered from three interconnected and interdependent perspectives: a conceptual, an empirical, and a technical level of investigation (Friedman et al., 2013). In an iterative and integrative process of ethical inquiry and interdisciplinary deliberation, this methodology is used to define values to be translated into the design by establishing standards and analyzing technical requirements that will guarantee the practical implementation of the values. In this line of argument, the focus is not on a retrospective ethical analysis but rather on proactively shaping and deliberating the development of a value sensitive design: Ethical and social considerations are incorporated into the design process and thus also develop the technical conditions for ethical development at an early stage. The advocates of this approach claim that human values are considered in “a principled and comprehensive manner” (Friedman et al., 2013, p. 55) throughout the design process.

Seeking to grasp how theory is understood, two premises must be explored: The basic premise of this approach is that technology is value laden. This means that human values and perceptions of values are incorporated into the design of an artifact before, during, or after the design process. Using methods such as stakeholder analyses, surveys, and feasibility studies, the VSD approach seeks to reveal the embodied and implicated values of the involved stakeholders. In this line of argument, exploring the inherent values of designs or artifacts is stated as relevant for individuals and society because values such as, for example, freedom, equality, trust, autonomy, and privacy are affected by technological processes and vice versa, as the advocates of this approach explain (Flanagan et al., 2008; Umbrello & Bellis, 2018). The other premise is that values are contextual and modified in the concrete field of application—the procedure is, therefore, “hardly deductive” (van de Poel, 2013). Hence, collective values and principles can only be implemented when proven to be relevant or in conflict in the tangible application situation. Therefore, the advocates of the VSD approach seek to incorporate value reflections into the design process in order to solve and address value problems at an early stage.

For the question of how to understand theory within VSD, these premises suggest that theory is mainly driven by a specific understanding of design. The design processes and their artifacts can be understood as the lynchpin of theory building due to their epistemological and moral potential. This means that within VSD approaches, it is assumed that both design processes and their artifacts generate new insights and knowledge in interaction with the humans in the loop. Specifically related to ethical theorizing, design processes and interactions with artifacts thus contribute to moral judgments and provoke, transport, or awaken certain value concepts. At first, this sounds very similar to the starting point of embedded ethics or ethically aligned approaches that also focus on the development process of AI. One difference, however, is the background of a profound design theory that assigns an inherent ethical significance to design. This marks clearly what design as a process or artifact already entails in terms of ethical implications. Thus, it is not only a development context in which ethical theory is situated but rather directly in the design as a process and artifact itself. This means ethical and moral judgments, potentials, and values are inherent in the design and become evident in interaction with designs. This slightly changes the perspective of ethical reflection and enriches it by taking into account on what ground the ethical reflection is taking place and what it is related to. Ethical theory building is, in this sense, a deeply context-sensitive but far more context-grounded endeavor. This is also the further unique selling point of the approach, in which interaction with design plays a central role and from which the interdisciplinary tripartite methodology with its empirical interest results.

The design with its moral and epistemological potential, subsequently, plays a central role for the understanding of practice within the VSD. Here, practice is understood as the interdisciplinary context-sensitive design process. Practice is not strictly separated as the opposite of theory, instead, ethical theory and design practices are connected. The tripartite methodology indicates this multidimensional understanding of practice that combines different conceptual, empirical, and technical perspectives of practice. Within this multidimensional approach, theory and practice are connected by their orientation toward the value-sensitive artifact as the objective of the design process. Practice is thus understood as the entirety of the contextual multidimensional design processes to produce an artifact. A value-sensitive artifact is, so to speak, condensed multidimensional practice.

As mentioned above, in the outline of the VSD approach, theory and practice are closely interwoven. The relation between theory and practice, however, can be defined even more concretely: in formulating design requirements, theory and practice merge. The conceptual locus of the interrelation between theory and practice affects the design requirements. As results of the design process and the ethical and scientific reflection by the involved stakeholders, the design requirements combine theoretical and practical ethical considerations. This means the practical conceptual conditions as well as ethical judgments shaped by design inherent values are formulated as contextual requirements, which are then further incorporated into the design process of a specific artifact.

The VSD methodology, however, is also subject to criticism (Jacobs & Huldtgren, 2018). One problem highlighted by criticisms is the identification of stakeholders (Manders-Huits, 2011). A quantitative study by Winkler and Spiekermann (2018) demonstrates that stakeholders are not identified accurately. This poses the fundamental question of whose values are addressed and heard in the design process. The problem of paternalistic judgments concerning values arises. The case of arbitrarily chosen values implies the limitations of this approach. Evaluation criteria and the reflection on generally applicable legal, social, and political standards and principles are missing. This points to a second criticism, namely that the concept of values remains underdetermined. Although Umbrello (2020) addresses this criticism by outlining an interpretation of values in terms of moral imagination theory, it remains ambiguous what kind of justifications exist for values and how they relate to overriding principles and ethical criteria. Manders-Huits (2011), furthermore, legitimately criticizes the lack of a methodology or theory of the VSD approach, especially in light of value conflicts. The conceptual absence of ethical-normative principles or evaluative criteria and their justification, moreover, determines a structural incapacity to discriminate between ‘good’ and ‘bad’ values, as well as to trade-off, or prioritize among incommensurable values in cases of conflict (Cenci & Cawthorne, 2020). The VSD methodology, thus, contains the potential for arbitrariness and corruption of ethical reflections if it does not rely on ethical-normative principles and justifications.

Umbrello and van de Poel (2021) address these conceptual shortcomings by linking them to another shortcoming of VSD, namely its lack of embeddedness in political and social contexts. They propose an orientation toward human rights to provide common guidance, especially in a globalized world. While human rights do provide a framework for ethical reflection, other values or principles (like well-being [Brey, 2015; Dennis, 2021], care [van Wynsberghe, 2013], or sustainability [van Wynsberghe, 2021]), however, may also be relevant in certain contexts. In Europe, for example, the ethical guidelines for trustworthy AI, accompanied by a political and legal process, provide principles that are far more context-specific than human rights (High-Level Expert Group on Artificial Intelligence, 2019). Moreover, the question arises as to how the political and social discourses can be embedded in the VSD approach to specify governance perspectives.

## A Meta-Framework for Applied AI Ethics Approaches

The analysis of these three approaches highlights the specific characteristics of each theory–practice conception with their guiding elements as well as their shortcomings (see Table 2): While the embedded ethics approach is driven by contextuality and, therefore, focuses on the situational ethical issues through stakeholder intuitions, ethically aligned approaches are guided by politically deliberated principles. The VSD approach, on the other hand, points out that the design process is a multidimensional endeavor of interdisciplinary deliberation and emphasizes design-inherent values. At the same time, however, the respective blind spots of the three approaches also reveal potentials how applied AI ethics approaches can conceptually bridge the gap between theory and practice: As we outlined in terms of the embedded ethics approach, we suggest the reflection of principles as an integral part of ethical decision-making and design to prevent subjectivity, arbitrariness, and power abuse. Regarding the ethically aligned conception, we highlighted the relevance of justifications of principles to justify ethical decision-making in a coherent and comprehensible way. Contextual values, moral intuitions, as well as technical knowledge and facts are combined and interdisciplinary deliberated in the VSD, yet the question of incorporating overarching principles and, furthermore, the question of addressing governance aspects arose. In Table 2, we summarize the guiding elements and the shortcomings of each conception.

**Table 2:** Guiding elements and shortcomings of the three analyzed AI ethics approaches: embedded ethics, ethically aligned approach, value sensitive design

Approach	Embedded ethics	Ethically aligned approach	Values sensitive design
Guiding elements	Stakeholder intuitions; Contextuality; Professional deliberation	Principles; Political deliberation	Interdisciplinary deliberation; Design inherent values
Shortcomings	Interrelation with overarching principles	Justification theories; Integration of stakeholder intuitions	Stakeholder identification; Value definition; Integration of overarching principles; Addressing governance aspects

**Fig. 1:**
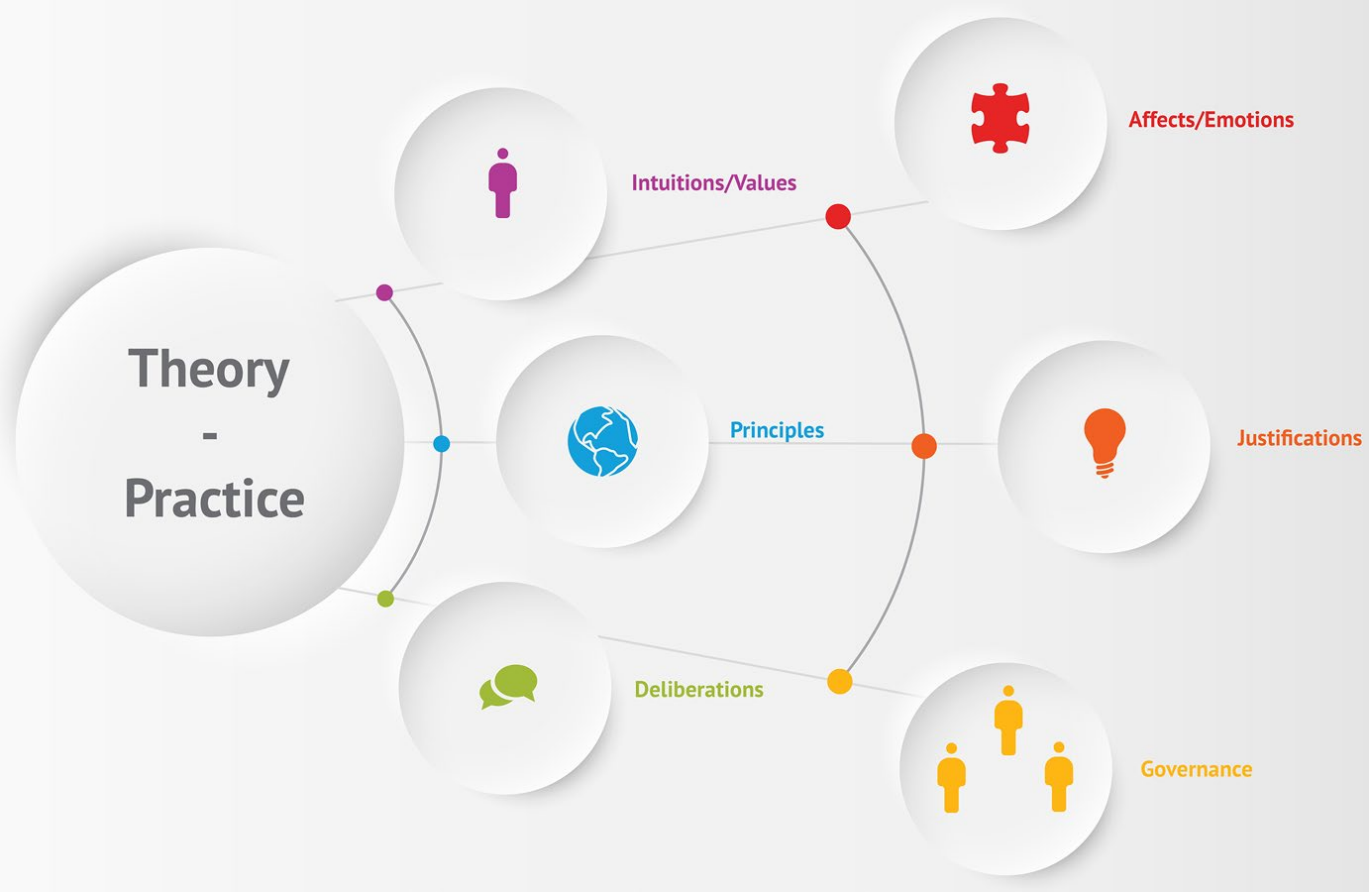
Meta-Framework for Applied AI Ethics Approaches and its Three Conceptual Dimensions The first dimension of reflection focuses on affects/emotions, the second dimension on justifications, and the third dimension asks about governance aspects. These dimensions are connected to the guiding elements that drive the three analyzed approaches: intuitions, principles, and deliberation

Against this background, we propose a meta-framework for applied AI ethics conceptions with three additional dimensions of ethical reflection (see Fig. 1). We do not claim that these three reflective dimensions are sufficient, but we do suggest that they might constitute starting points for critically reflecting current AI ethical conceptualizations of theory and practice. The aim is to address and overcome their blind spots. First, we suggest *affects and emotions* as elementary dimension to be integrated into AI ethical theory–practice conceptualizations. Second, the inclusion of *justifications* is important to negotiate competing principles. Third, the consideration of *governance* aspects extends the AI ethics’ understanding of practice and is crucial to keep in mind the political, legal, and social conditions, implications, and courses of action relevant to theory–practice considerations and, if necessary, to address them at a political level. This meta-framework is intended as a reflective tool for understanding, mapping, and assessing applied AI ethics approaches.

Based on critical theory (Waelen, 2022), we argue that the mentioned three dimensions provide important conceptual perspectives to identify blind spots and shortcomings of AI ethics approaches in terms of a theory–practice gap. The critical-theoretical approach is instructive insofar as it theorizes how theory is formed through practice. Theory and practice are, accordingly, not trapped in a strict or perceived dichotomy; rather, theory is shaped by the experiences of practice and vice versa. A critical theory approach also focuses on the possibilities of empowering individuals in the face of power asymmetries and marginalization. Critical theory is thus particularly sensitive to power relations and experiences of injustice and disregard. In that sense, the approach in this paper follows a vulnerability-sensitive and -theoretical approach toward ethical theory building. In terms of critical theory, therefore, these three dimensions concern the questions of whose voices are heard, which power structures are at work, and how practice is understood.

The first dimension is proposed to capture the entangled *affects and emotions* of all agents involved in or affected by concrete ethical decision-making. Such affects and emotions are important because they can provide insights into overarching societal expectations, hazards, or experiences of inequity. At the same time, however, two central challenges relate to this: In different ethical theories, it still seems to be difficult to include emotions and affects in the formation of ethical judgments, especially when these are not attributable to individual entities but are located collectively. One reason for this is that the branch of empirical ethics is not only a comparatively young branch of research within ethics, but at the same time, there is great uncertainty about which normative content can be assigned to descriptive assessments. A second problem is that we still do not sufficiently understand the significance of affects and emotions for the formation of ethical judgments. Of course, the reference to intuitions and individual or collective value conceptions as expressed in the VSD or embedded ethics approach is crucial. But at the same time, the central question is whose intuitions are being taken into account here: Various scholars and studies have pointed out that, especially with regard to the intuitions and values taken into account, it is the values of certain—mostly white and male—persons that are considered to be relevant (Cave & Dihal, 2020; Mohamed et al., 2020). The systematical exploration of articulated affects and emotions and their incorporation into ethical decision-making then represents a way of integrating experiences of disregard and marginalization into both the description of applied practices and the critical examination of considered intuitions.

*Participatory elements* also provide access to the dimension of affects and emotions of the involved agents and groups. Participation can look very different: On the one hand, it can mean opening up the engineering process in the sense of an open innovation or a citizen science approach and letting individuals actively participate in it. On the other hand, it can also mean empirically evaluating concrete user experiences, moral attitudes, and attitudes toward certain technologies. It is also debatable whether participation can perhaps be achieved via information and science communication by means of transparency requirements. The fact that participation always involves exclusion, however, must be taken into account and addressed accordingly if, for example, a biased representation emerges. Both passive and active elements of participation are conceivable and need to be examined within applied AI ethics approaches. The VSD method has, in this regard, the potential to take the lead in these participatory efforts, or at least be complemented by participatory elements: the VSD method is based, on the one hand, on the inclusion of all stakeholders and, on the other hand, has already been the subject of criticism in terms of stakeholder identification. Perhaps, considering this criticism, a participatory perspective offers new opportunities for methodological adjustments to VSD.

A second dimension is suggested to identify the *justifying normative background theories* of the principles chosen. Why is this crucial? Several studies, in particular those by Jobin et al. (2019) and Hagendorff (2020), show that in different reports and governance approaches as well as in different ethical papers, the principles considered to be central vary greatly. Which principles, however, are selected and why it is exactly these and not others, depends decisively on the reasons chosen. Whether the principle of justice is seen as a central principle for AI ethics or not changes, on the one hand, the kind of normative claims that are considered relevant. At the same time, the way the principles are weighed is not simply neutral but can either cement power relations or question existing power relations. This is by no means a new point. In the context of medical ethics, this has been discussed for quite a long time. But interestingly, it still does not yet play a central role in the analyzed AI ethics approaches. Including the dimension of justifications creates two advantages: It is no longer possible without further ado to hide behind a principle that is regarded as established without, at the same time, having to justify why exactly this principle is ascribed a normative orienting power. Negotiating rationales also leads to a stronger debate about what can and should be the aim and task of applied AI ethics.

Especially in politically turbulent times, in times when the self-evidence of social cohesion seems fragile and forms of common action are urgently needed, it is of central importance to explain and to justify why certain principles are considered supportive, which principles may be added, and which principles are no longer considered beneficent. This is especially of interest when principles conflict with each other, for instance, when the autonomy of the individual is weighed against ideas of the common good. Here, a decision or ranking in favor of one principle or the other can only be made if reasons are provided as to why autonomy prevails in certain cases, why transparency is preferable to economic interests, or why the safety of technology is more important than absolute transparency.

In concrete terms, the issue of the justifications of principles also depends on the *context*. Context sensitivity, therefore, is relevant for developing coherent justification theories for principles. A human-rights oriented AI ethics approach, for example, may be justified in a medical context on the grounds of patient well-being, whereas in the context of AI driven automated legal decision-making it is more likely to protect against injustice, discrimination, or marginalization. The embedded ethics approach provides in this respect a decisive focus; the conceptual embedding and associated justification motives are the litmus test for ethical principles.

A third dimension considers *governance aspects* of design. This aspect is particularly important because, in all approaches, the social, political, and legal discourses seem to be only loosely connected to their understanding of practice. However, governance in the sense of bringing together issues of hard law, soft law, participation, empowerment, and IT-design may in fact function as a binding factor for realizing an ethical AI design and its application. This third dimension offers the potential to reflect on practice in a broader understanding and contribute to political discourses, considering social, political, and legal conditions and addressing the public discourse on AI with solutions or contributions to governance aspects. For this purpose, the governance lens helps to develop and apply AI in terms of its embeddedness into social and political structures and practices. In addition, it provides insights into societally and politically developed norms and principles for ethical considerations. The question to be raised is, consequently: Which legal regulations, social norms, and political discussions should be considered and included in the ethical AI engineering process? This is by no means an abstract question but can be seen concretely in the current debates about regulatory approaches to artificial intelligence, such as the European debates about the AI Act or the framework of the Ad Hoc Committee on Artificial Intelligence (CAHAI). The shift from an (individual-) rights-oriented governance to a risk-based AI governance has direct consequences on how far specific needs of persons with incapacities can be considered or not, for instance. The moment ethical principles such as vulnerability (Bleher & Braun, 2022; Braun, 2020) or justice (Braun & Hummel, 2022; Braun et al. 2021) are justified as normatively central, the evaluation of primarily risk-based governance of AI systems also changes.

In this sense, the governance dimension focuses on *structural empowerment*. Not only the embedding in structures but also the creation of new structures is relevant in this dimension. An ethically aligned approach basically engages in such a structural perspective when concrete politically shaped principles are linked to tools or mechanisms and design requirements or considerations. Given political structures are thus translated into technical strategies, in other words, structures are analyzed in terms of possibilities for action. How structures are designed, however, depends not least on the question of who is affected by what structures at which level and who has the possibility to change them. In this context, it is not only relevant to analyze which micro-, meso-, or macro-structures are at work in the context of AI engineering but also to ask who is empowered and has the possibilities to address or change structures. In concrete terms, this could imply seeking opportunities and possibilities in the ethical AI engineering process to enable the transformation of structures for the development of ethical AI and to empower individuals to do so.

## Conclusion

This article addresses the issue of conceptualizing theory and practice in applied AI ethics approaches. The guiding question was how to mediate between the *what* and the *how* of AI ethics. Three exemplary applied AI ethics approaches served as references to explore this question. Therefore, we hermeneutically analyzed the understanding of theory and practice as well as their entanglement in each approach. The different notions of theory and practice revealed the distinctive emphases and the guiding element of each conception. Moreover, delineating the specific potentialities and shortcomings of the approaches rendered the conceptualization of theory and practice a multi-faceted endeavor. Against this background, we have proposed three reflective dimensions: first, affects and emotions; second, justifications; and third, governance aspects. We argue in terms of critical theory that these dimensions provide a meta-framework understood as a reflective tool to understand, map, and assess current applied AI ethics conceptualizations of theory and practice. This meta-framework aims to address and overcome the blind spots of these theory–practice conceptualizations by reflecting on experiences of discrimination and marginalization, power structures, and the embeddedness of digital practices in political discourses.
